# Do the new triatomine species pose new challenges or strategies for monitoring Chagas disease? An overview from 1979-2021

**DOI:** 10.1590/0074-02760210015

**Published:** 2021-05-31

**Authors:** Jane Costa, Carolina Dale, Cleber Galvão, Carlos Eduardo Almeida, Jean Pierre Dujardin

**Affiliations:** 1 Fundação Oswaldo Cruz-Fiocruz, Instituto Oswaldo Cruz, Laboratório de Biodiversidade Entomológica, Rio de Janeiro, RJ, Brasil; 2 Fundação Oswaldo Cruz-Fiocruz, Instituto Oswaldo Cruz, Laboratório Nacional e Internacional de Referência em Taxonomia de Triatomíneos, Rio de Janeiro, RJ, Brasil; 3 Universidade Federal da Bahia, Salvador, BA, Brasil; 4 Universidade Estadual de Campinas, Instituto de Biologia, Campinas, SP, Brasil; 5 Institut de Recherche pour le Développement, Montpellier, France

**Keywords:** American Trypanosomiasis, vectors, Triatominae subfamily

## Abstract

Chagas disease persists as one of the most important, and yet most neglected, diseases in the world, and several changes in its epidemiological aspects have been recorded since its discovery. Currently, some of the most relevant changes are related to: (i) the reduction in the incidence of the endemic due to the control of the most important vectors, *Triatoma infestans* and *Rhodnius prolixus*, in many countries; (ii) the migration of human populations spreading cases of the disease throughout the world, from endemic to non-endemic areas, transforming Chagas disease into a global threat; and (iii) new acute cases and deaths caused by oral transmission, especially in the north of Brazil. Despite the reduction in the number of cases, new challenges need to be responded to, including monitoring and control activities aiming to prevent house infestation by the secondary vectors from occurring. In 1979, Lent & Wygodzinsky^(1)^ published the most complete review of the subfamily Triatominae, encompassing 111 recognised species in the taxon. Forty-two years later, 46 new species and one subspecies have been described or revalidated. Here we summarise the new species and contextualise them regarding their ecology, epidemiologic importance, and the obstacles they pose to the control of Chagas disease around the world.

Despite the great achievements in controlling Chagas disease, also known as American Trypanosomiasis, major problems are still to be solved in Latin American countries.^2^ No vaccines or drugs are currently available to cure the chronic phase of this disease that affects six million people around the world and has its epidemiology constantly changing because of ecological, climatic, social, political, and technical factors.^3^ ^,^ ^4^ ^,^ ^5^ ^,^ ^6^

The most effective action in terms of Chagas disease control is the elimination of its vectors from the human dwellings^7^ and, as a means to achieve this goal, four multigovernmental initiatives were launched (South America, Andean countries, Mexico and Central America, Amazon) targeting the three most important triatomine species that transmit the etiological agent *Trypanosoma cruzi*: *Triatoma infestans* (Klug, 1834) (southern South America), *Rhodnius prolixus* Stål, 1859, and *T. dimidiata* (Latreille, 1811) (northern South America and Central America). The most remarkable achievement resulting from these initiatives was the elimination of the *T. infestans* domiciliated populations in Brazil, Chile, Uruguay, various provinces in Argentina, and several regions in Paraguay.^8^ More recently, the elimination of the domiciliary infestations of *R. prolixus* from Central America.^9^ ^,^ ^10^ has also been considered a significant accomplishment regarding the battle against vectorial Chagas disease transmission.

Carlos Chagas^11^ described the disease in 1909, when the great majority of cases were due to vectorial transmission by the triatomine bug *T. infestans*, the species most well adapted to human dwellings in many countries in Latin America*.* Later, several other possibilities of transmission were attested, such as those observed as the result of the donation of infected blood or organs, or the ingestion of contaminated food, mother-child transmission and because of laboratory work accidents.^12^ ^,^ ^13^ ^,^ ^14^

More than 10 years after the certification of the elimination of the vectorial transmission by *T. infestans* in some countries, the illness remains as one of the most important neglected diseases and is now spreading into some non-endemic areas because of human migrations.^15^ ^,^ ^16^ Countries such as Australia, Canada, Japan, Spain, and the United States of America are the most affected by immigrants infected by *T. cruzi*.^17^ ^,^ ^18^ ^,^ ^19^ ^,^ ^20^ ^,^ ^21^ ^,^ ^22^ ^,^ ^23^ Furthermore, climatic and environmental changes may develop new behavioral patterns and adaptations of the triatomines, causing unexpected occurrences of transmission to be recorded.^24^ Oral transmission, for instance, has been causing new acute cases and deaths, especially in the North of Brazil and Venezuela.^25^ ^,^ ^26^ ^,^ ^27^ An important factor of oral transmission is the proximity of humans to infected vectors. Therefore, vectors also represent a key-factor for this kind of contamination. According to Dias et al.,^28^ new epidemiological situations have been observed in the last years. *T. infestans* has been eliminated in large geographic areas but remains infesting natural and artificial ecotopes in the Chaco region, especially in Argentina,^29^ while a similar situation can be observed in northern South and Central America regarding *R. prolixus*.^9^ Additionally, the invasion of houses by *T. tibiamaculata* (Pinto, 1926) in Salvador (Bahia, Brazil) also stresses the importance of continuously monitoring the vectors.^30^ In conclusion, understanding the biology and ecology of the triatomines and their associations with humans is crucial to avoid new cases of Chagas disease.^31^ ^,^ ^32^ ^,^ ^33^ ^,^ ^34^

The objective of this review is to summarise the new species described after 1979, when Lent & Wygodzinsky^1^ published the most complete review about the Triatominae subfamily. In this review we contextualise the new vector species regarding their ecological characteristics, epidemiologic importance, and the new obstacles they pose to the monitoring and control of Chagas disease around the world.

Triatominae until 1979

Triatomines have been known since the 18th century, when the first species, *T. rubrofasciata* (De Geer, 1773), first called “*Cimex rubrofasciatus*” (De Geer, 1773), was described in Indonesia. At that time, this tropicopolitan species was not recognised as a potential vector. Several other Triatominae species had been described before the discovery of the disease, such as *T. infestans* and *Panstrongylus megistus* (Burmeister, 1835); the latter being the first species to be shown as a vector in the endemic areas of the State of Minas Gerais, Brazil. However, 136 years passed after the description of *T. rubrofasciata* until the disease was described by Carlos Chagas. Despite the difficulties in proving the existence of the new disease in a region plagued by a great number of other illnesses, the Brazilian physicist Carlos Chagas described not only its symptoms and the clinical aspects, but also the sensitive agents, the hosts, the etiologic agent, and the vectors.^11^ ^,^ ^35^ ^,^ ^36^

The great majority of the bugs belonging to this subfamily, such as *Cavernicola lenti* Barrett & Arias, 1985, *C. pilosa* Barber, 1937, *Psammolestes coreodes* Bergroth, 1911, *P. tertius* Lent & Jurberg, 1965, *Parabelminus carioca* Lent, 1943, and *P. yurupucu* Lent & Wygodzinsky, 1979, among many others, can only be found in their natural sylvatic environments.^1^ Many other species are able to eventually invade domiciles, mainly attracted by light,^37^ but just a few species are recognised as major threats to public health, like *T. brasiliensis brasiliensis* Neiva, 1911, *T. infestans*, *T. pseudomaculata* Corrêa & Espínola, 1964, *T. sordida* (Stål, 1859), *P. megistus*, *R. prolixus*, *R. ecuadoriensis* Lent & Leon, 1958, and *T. dimidiata*.^38^ Thus, the knowledge about the process of adaptation of the triatomines to the human dwellings is fundamental to design and propose effective vector control strategies.^39^

After the discovery of the disease, Neiva^40^ was one of the first scientists interested in understanding the vectorial capacity of the distinct species, and Lent & Wygodzinsky^1^ have published the most complete review of the triatomines so far.

Target species

As previously mentioned, despite the achievements in controlling populations of the most important anthropophilic vector, several native species must be monitored, reaffirming the need for constant technical activities to check for invasions or domiciliation. This group of species includes: *T. brasiliensis*, *P. geniculatus* (Latreille, 1811), *R. brethesi* Matta, 1919, *R. prolixus*, *R. nasutus* Stål, 1859, *R. neglectus* Lent, 1954, *T. dimidiata*, *T. maculata* (Erichson, 1848), *T. rubrovaria* (Blanchard, 1843), *T. sanguisuga* (Leconte, 1855), and *T. vitticeps* (Stål, 1859) according to Costa & Lorenzo.^33^

*Triatoma rubrofasciata* represents a particular case. This species exhibits domestic habits and is found predominantly in the New World, mainly in Latin America. Although it has only been reported in very limited regions of Brazil in the past 30 years, there have been occurrences of the species feeding on humans in Southeast Asia, where severe bite reactions, sometimes leading to anaphylactic shock, have been recorded. Reports of this species in Asia have increased significantly in the last five years.^41^ ^,^ ^42^ Recently, this species has also been recorded in China.^43^ ^,^ ^44^ Thus, its epidemiological importance is a matter of concern.^45^ The trend to increased domesticity of this species represents what we could expect from other species which are not closely monitored because they are not currently regarded as target species.

Native vectors

More than ten different species of triatomines have been found in the United States of America, with the highest diversity and density in the states of Texas, New Mexico, and Arizona.^46^ In Texas and other Southern states of the USA, the most collected species were *T. sanguisuga* and *T. gerstaeckeri* (Stål, 1859). These native vectors are occasionally found in households, but usually without robust signs of colonisation.^47^

*Rhodnius prolixus* is the main Chagas disease vector in Venezuela, Colombia, and certain areas of Central America, where it can build up large colonies inside human domiciles.^48^ The other two main species implicated in Chagas disease eco-epidemiology in Central American countries are *T. dimidiata* and *R. pallescens* Barber, 1932.^49^ The members of the *T. phyllosoma* complex (Meccus Stål, 1859 in some literature) are also found invading and colonising human domiciles in Mexico.^18^

The current scenario is quite challenging in Brazil, where there are 66 triatomine species recorded, of which 37 are native. Therefore, the country presents the highest diversity in this group of insects.^33^ ^,^ ^34^ ^,^ ^50^ ^,^ ^51^ ^,^ ^52^ ^,^ ^53^ ^,^ ^54^

It is also important to highlight that more than 20 triatomine species have already been recorded in the Brazilian Amazon Forest,^55^ ^,^ ^56^ ^,^ ^57^ ^,^ ^58^ ^,^ ^59^ ^,^ ^60^ which corresponds to roughly 40% of the Brazilian territory and is one of the richest areas on the planet in terms of biodiversity.^61^ Some of the difficulties found in this region are: (i) the lack of data on the habitats of the newly described triatomines; (ii) triatomines that may be losing their natural habitats because of environmental changes; (iii) the very probable existence of undescribed species; and (iv) the lack of detailed studies on the species already recorded in the area. These factors impede an accurate estimate of the risk of dissemination of the Chagas disease in the Amazon.^62^

Triatominae after 1979

Since the publication of the remarkable Lent & Wygodzinsky^1^ monograph in 1979, describing and illustrating 111 triatomines, 46 species and one subspecies have been described as new or revalidated. They were included in 12 of the 19 genera of the subfamily, which now represent 157 known species (154 living species and three fossils) from 15 countries,^52^ ^,^ ^53^ ^,^ ^63^ ^,^ ^64^ ^,^ ^65^ ^,^ ^66^ ^,^ ^67^ ^,^ ^68^ plus a subspecies^69^ (Table). Out of those 47 triatomines, 17 are from Brazil, followed by Mexico and Colombia, each with four species (post-1979). In the remainder 12 countries, 22 triatomines have been recorded, and the numbers varied from one to two in each one (Table, Figure).

Twenty-one of the 47 new or revalidated taxa (post-1979) belong to the genus *Triatoma* Laporte, 1832, nine to *Rhodnius* Stål, 1859, and four to *Panstrongylus* Berg, 1879 (Table). The fact that 34 of the 47 newly validated triatomines belong to the three genera with the highest medical importance is noteworthy. However, among them, only five show clues of house invasion or domiciliation. The three first triatomines, *T. juazeirensis* Costa & Felix, 2007, *T. b. macromelasoma* Galvão, 1956, and *T. sherlocki* Papa et al., 2002, are included in the *T. brasiliensis* species complex.^69^ ^,^ ^70^ ^,^ ^71^ *Triatoma bahiensis* Sherlock & Serafim, 1967 and *T. melanica* Neiva & Lent, 1941 were also included in that species complex and eventually invade houses, but have not exhibited signals of domiciliation yet.^37^ ^,^ ^71^ ^,^ ^72^ ^,^ ^73^ The fourth triatomine, *T. rosai* Alevi et al. is able to colonise a great diversity of natural ecotopes and is also found infesting domiciliary and peridomiciliary areas in Argentina, as well as in Bolívia and Paraguay.^68^ ^,^ ^74^ The fifth species, *T. huehuetenanguensis* was found naturally infected by *T. cruzi* in domestic ecotopes.^65^ The two exceptions of triatomines (Table) collected in the intradomicile without clues of domiciliation or frequent invasion are the *Belminus* species, *B. corredori* and *B. ferroae*, known to be sylvatic species. Both were captured in Colombia, inside dwellings.^75^ ^,^ ^76^ Since then, no further report on these species in domiciliary ecotopes have been made. Therefore, it is highly probable that those specimens invaded the houses when they were captured.

**TABLE:** Species published after Lent & Wygodzinsky,^(1)^ based on Galvão et al.^(50)^ and updated. Type localities are marked in the map according to the numbers in parentheses.

Genus	Species	Author	Year	Type locality	Distribution	Collection (Type)	Ecotope	DNA sequence
*Alberprosenia*	*malheiroi*	Serra, Atzingen & Serra	1987	Jacundá (1)	Pará, Brazil	Faculdade de Saúde Pública, Universidade de São Paulo, São Paulo, Brazil	*Oenocarpus bacaba*	-
*Belminus*	*corredori*	Galvão & Angulo	2006	San Gil (2)	Santander, Colombia	Herman Lent Collection, Instituto Oswaldo Cruz, Rio de Janeiro, Brazil	Dwellings	-
	*ferroae*	Sandoval, Pabón, Jurberg & Galvão	2007	Toledo (3)	North Santander, Colombia	Herman Lent Collection, Instituto Oswaldo Cruz, Rio de Janeiro, Brazil	Dwellings	-
	*laportei*	Lent, Jurberg & Carcavallo	1995	Utinga (4)	Pará, Brazil	Herman Lent Collection, Instituto Oswaldo Cruz, Rio de Janeiro, Brazil	-	-
	*pittieri*	Osuna & Ayala	1993	Rancho Grande (5)	Aragua, Venezuela	Colección de Insectos, Francisco Fernandéz Yépez del Museo del Instituto de Zoologia Agricola (MIZA)	High altitude	-
*Cavernicola*	*lenti*	Barrett & Arias	1985	Balbina (Hidroeletric) (6)	Amazon, Brazil	Herman Lent Collection, Instituto Oswaldo Cruz, Rio de Janeiro, Brazil	Hollow tree	18S
*Hermanlentia*	*matsunoi*	(Fernández-Loayza)	1989	Pias (7)	Pataz, Peru	Herman Lent Collection, Instituto Oswaldo Cruz, Rio de Janeiro, Brazil	-	-
*Linshcosteus*	*karupus*	Galvão, Patterson, Rocha & Jurberg	2002	Kalakkadu (8)	Tamil Nadu State, India	Herman Lent Collection, Instituto Oswaldo Cruz, Rio de Janeiro, Brazil	Rock formation	28S, 16S, 18S
*Meccus*	*bassolsae*	(Alejandre-Aguilar et al.)	1999	San Jeronimo, Xayacatlán Acatlán (9)	Puebla, Mexico	Collection of Parasitology Department in ENCB-IPN, Mexico	-	Cytb, 28S
*Mepraia*	*gajardoi*	Frias, Henry & González	1998	Arica (10)	Caleta Vitor, Chile	Insect Collection of the Instituto de Entomologia, Universidad Metropolitana de Ciencias de la Educacion, Santiago, Chile	Coastal desert	Cytb, COI
	*parapatrica*	Frías	2010	Pan de Azúcar National Park (11)	Atacama, Chile	Collection of the Institute of Entomology, Universidad Metropolitana de Ciencias de la Educación (IEUMCE), Santiago, Chile	Coastal area	Cytb, COI
*Nesotriatoma*	*confusa*	Oliveira, Ayala, Justi, Rosa & Galvão	2018	- (12)	Cuba	Herman Lent Collection, Instituto Oswaldo Cruz, Rio de Janeiro, Brazil	-	-
*Paleotriatoma*	*metaxytaxa* ^*^	Ponair	2019	Hukawng Valley (13)	Kachin, Myanmar	Ponair Ambar collection, Oregon State Univeristy	Ambar	-
*Panstrongylus*	*hispaniolae* ^*^	Ponair	2013	La Toca Amber mine, Cordillera Septentrional (14)	Dominican Republic	Ponair Ambar Collection, Oregon State University	Ambar	-
	*martinezorum*	Ayala	2009	Cataniapo River (15)	Puerto Ayacucho, Venezuela	Museum of Instituto de Zoología Agrícola Francisco Fernández Yépez (MIZA), Universidad Central de Venezuela, Maracay	-	-
	*mitarakaensis*	Bérenger & Blanchet	2007	Border of French Guiana with Brazil (16)	French Guiana	Department of Hemiptera, Museum National d’Histoire Naturelle, Paris, France	-	-
	*sherlocki*	Jurberg, Carcavallo & Lent	2001	Santo Inácio (17)	Bahia, Brazil	Rodolfo Carcavallo Collection, Instituto Oswaldo Cruz, Rio de Janeiro, Brazil	200-500m altitude	-
*Rhodnius*	*amazonicus*	Almeida, Santos & Sposina	1973	Itacoatiara (18)	Amazon, Brazil; French Guiana (Cacao, Saül)	INPA, Instituto Nacional de Pesquisas da Amazônia, Manaus, Brazil	*-*	-
	*barretti*	Abad-Franch, Palomeque & Monteiro	2013	Puerto Asís (19)	Departament of Putumayo, Colombia; Sucumbíos province, Ecuador	Herman Lent Collection, Instituto Oswaldo Cruz, Rio de Janeiro, Brazil	*Attalea butyracea* and *Oenocarpus bataua*	-
	*colombiensis*	Mejia, Galvão & Jurberg	1999	Totarco (20)	Coyaima, Colombia	Herman Lent Collection, Instituto Oswaldo Cruz, Rio de Janeiro, Brazil	*Attalea butyracea*	Cytb, 16S, 18S
	*marabensis*	Souza et al.	2016	Marabá (21)	Pará, Brazil	N. C. B. Von Atzingen, M. B. Furtado UNESP	Dwellings (Murumurú Environmental Reserve)	Cytb
	*micki*	Zhao, Galvão & Cai	2021	Santa Cruz, Saavedra (22)	Bolivia	Natural History Museum, UK	*-*	-
	*milesi*	Carcavallo, Rocha, Galvão & Jurberg	2001	Bragança (23)	Pará, Brazil	Rodolfo Carcavallo Collection, Instituto Oswaldo Cruz, Rio de Janeiro, Brazil	*Maximilian regia* and *Attalea speciosa*	-
	*montenegrensis*	Rosa et al.	2012	Monte Negro (24)	Rondonia, Brazil	Herman Lent Collection, Instituto Oswaldo Cruz, Rio de Janeiro, Brazil	*Orbignya phalerata*	Cytb
	*stali*	Lent, Jurberg & Galvão	1993	Salobra (25)	Mato Grosso, Brazil	Herman Lent Collection, Instituto Oswaldo Cruz, Rio de Janeiro, Brazil	*Attalea phalerata*	Cytb, 16S, 18S
	*zeledoni*	Jurberg, Rocha & Galvão	2009	Aracaju (26)	Sergipe, Brazil	Herman Lent Collection, Instituto Oswaldo Cruz, Rio de Janeiro, Brazil	-	-
*Triatoma*	*bahiensis* ^**^	Sherlock & Serafim	1967	Ipupiara (27)	Bahia, Brazil	Herman Lent Collection, Instituto Oswaldo Cruz, Rio de Janeiro, Brazil	-	Cytb
	*baratai*	Carcavallo & Jurberg	2002	Bonito (28)	Mato Grosso, Brazil	Rodolfo Carcavallo Collection, Instituto Oswaldo Cruz, Rio de Janeiro, Brazil	Near a cave	Cytb, COI, 16S
	*bolivari*	Carcavallo, Martínez & Pelaez	1987	Colima (29)	Jalisco, Mexico	Rodolfo Carcavallo Collection, Instituto Oswaldo Cruz, Rio de Janeiro, Brazil	-	Cytb, ITS-2
	*boliviana*	Avendaño, Espada, Gil, Asturizaga, Mamani & Prieto	2007	Muñecas (30)	La Paz, Bolivia	Colección Boliviana de Fauna del MNHN, Facultad de Ciencias Puras y Naturales de la Univ. Mayor de SanAndrés, La Paz, Bolivia	Rocks	-
	*brailovskyi*	Martínez, Carcavallo & Pelaez	1984	Colima (31)	Jalisco, Mexico	Rodolfo Carcavallo Collection, Instituto Oswaldo Cruz, Rio de Janeiro, Brazil	-	Cytb, ITS-2
	*brasiliensis macromelasoma* ^**^	Galvão	1956	Petrolina (32)	Pernambuco, Brazil	Instituto Oswaldo Cruz Entomological Collection, Rio de Janeiro, Brazil	-	Cytb
	*carcavalloi*	Jurberg, Rocha & Lent	1998	Santana do Livramento (33)	Rio Grande do Sul, Brazil	Herman Lent Collection, Instituto Oswaldo Cruz, Rio de Janeiro, Brazil	Under rocks	Cytb, COI, COII, 16S, 18S
	*dominicana* ^*^	Ponair	2005	La Toca Amber mine, Puerto Plata and Santiago (34)	Dominican Republic	Ponair Ambar Collection, Oregon State University	Ambar	-
	*garciabesi* ^**^	Carcavallo, Cichero, Martínez, Prosen & Ronderos	1967	Córdoba (35)	Argentina	Rodolfo Carcavallo Collection, Instituto Oswaldo Cruz, Rio de Janeiro, Brazil	-	-
	*gomeznunezi*	Martínez, Carcavallo & Jurberg	1994	Portillo del Rayo (36)	Oaxaca, Mexico	Rodolfo Carcavallo Collection, Instituto Oswaldo Cruz, Rio de Janeiro, Brazil	Under rocks	-
	*huehuetenanguensis*	Lima-Cordón & Justi	2019	Huehuetenango (37)	Aldea Chamuxu, Guatemala	Instituto Oswaldo Cruz Entomological Collection, Rio de Janeiro, Brazil	Domiciliary ecotopes	ITS-2 Cytb
	*jatai*	Gonçalves, Teves-Neves, Santos-Mallet, Carbajal-de-la-Fuente & Lopes	2013	Fazenda Jataí (38)	Tocantins, Brazil	Herman Lent Collection, Instituto Oswaldo Cruz, Rio de Janeiro, Brazil	Rock outcrops	-
	*juazeirensis*	Costa & Felix	2007	Juazeiro (39)	Bahia, Brazil	Instituto Oswaldo Cruz Entomological Collection, Rio de Janeiro, Brazil	-	Cytb, ITS-1 ITS-2
	*jurbergi*	Carcavallo, Galvão & Lent	1998	Rondonópolis (40)	Mato Grosso, Brazil	Rodolfo Carcavallo Collection, Instituto Oswaldo Cruz, Rio de Janeiro, Brazil	-	Cytb, COI, 16S, 18S
	*klugi*	Carcavallo, Jurberg, Lent & Galvão	2001	Nova Petrópolis (41)	Rio Grande do Sul, Brazil	Rodolfo Carcavallo Collection, Instituto Oswaldo Cruz, Rio de Janeiro, Brazil	Rock crevices	Cytb, COI, COII, 16S
	*melanica* ^**^	Neiva & Lent	1941	Espinosa (42)	Minas Gerais, Brazil	Instituto Oswaldo Cruz Entomological Collection, Rio de Janeiro, Brazil	-	Cytb
	*mopan*	Dorn, Justi & Dale	2018	Rio Frio cave (43)	Cayo, Belize	Instituto Oswaldo Cruz Entomological Collection, Rio de Janeiro, Brazil	Cave	ITS-2 Cytb
	*pintodiasi*	Jurberg, Cunha & Rocha	2013	Vila Nova do Sul (44)	Rio Grande do Sul, Brazil	Herman Lent Collection, Instituto Oswaldo Cruz, Rio de Janeiro, Brazil	Under rocks	-
	*rosai*	Alevi *et al.*	2020	San Miguel (45)	Corrientes, Argentina	Dr. Jose Maria Soares Barata Triatominae Collection (CTJMSB) of the São Paulo State University	Fallen trunks, tree holes, bromeliads, palm trees, in opossum holes and in dry cacti, and domiciliary ecotopes	Cytb
	*sherlocki*	Papa, Jurberg, Carcavallo, Cerqueira & Barata	2002	Santo Inácio (46)	Bahia, Brazil	Faculdade de Saúde Pública, Universidade de São Paulo, São Paulo, Brazil	Rocks	Cytb, COI, COII, 16S, 28S
	*vandae*	Carcavallo, Jurberg, Rocha, Galvão, Noireau & Lent	2002	Itiquira (47)	Mato Grosso, Brazil	Rodolfo Carcavallo Collection, Instituto Oswaldo Cruz, Rio de Janeiro, Brazil	Stone walls	Cytb, COI, COII, 16S, 18S, 28S

*: fossil species; **: revalidated after 1979.

**Figure:**
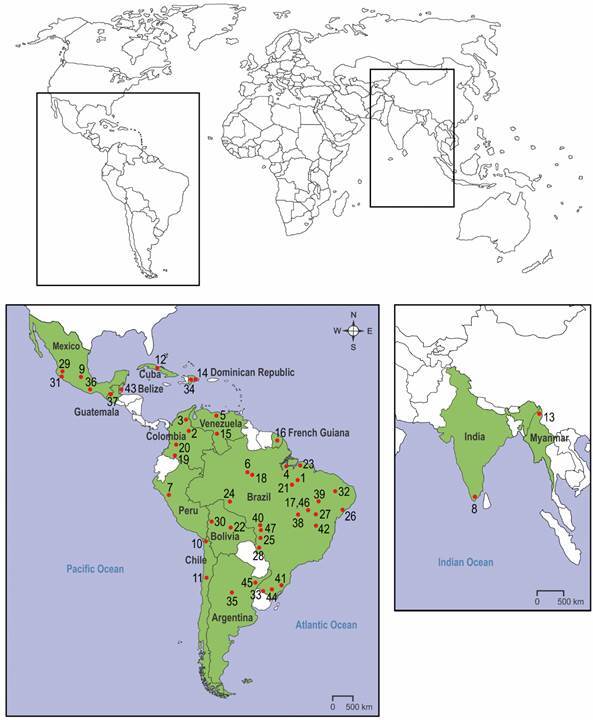
Distributional map of the 46 Triatominae species and one subspecies described or revalidated after Lent & Wygodzinsky,^(1)^ from 15 countries and three continents. Numbers represent the type localities listed in the table.

In this sense, the great majority, 42 of the 47 triatomines listed post-1979, do not appear to be capable of changing the epidemiology or the currently known *T. cruzi* transmission profiles to human populations (Table).

Valid species and the importance of integrative analysis

*Rhodnius amazonicus* Almeida, Santos & Sposina, 1973 and *R. zeledoni* Jurberg Rocha & Galvão, 2009 are triatomines of rare occurrence, recorded in the north and northeastern Brazil.^77^ ^,^ ^78^ However, they still need to have their taxonomic status confirmed molecularly.^79^ It is applied also to a Bolivian species - *T. boliviana* Martinez et al., 2007, related to *T. nigromaculata* (Stål, 1859).^80^ The confirmation of the specific status of species of the genus *Rhodnius* by means of molecular data and experimental crossings proved to be extremely important in the face of the events of cryptic speciation and phenotypic plasticity of the species.^66^ ^,^ ^79^ *Rhodnius taquarussuensis* Rosa et al., 2017, for example, was recently synonymised with *R. neglectus* after applying molecular analyses.^66^ *T. rosai* is closely related to *T. sordida* and was characterised based on integrative taxonomy which is crucial for describing and characterising new taxa. Unfortunately, at times, the integrative taxonomy cannot be carried out as is the case of the recently described species *R. micki* Zhao, Galvão & Cai, 2021. Its characterisation was based on morphological and geometric morphometrics analysis using specimens from a collection.^81^

In regard to the Triatominae, phenotypic variability has been observed in several taxa, so the multidisciplinary approach is mandatory to avoid misinterpretation of the intra specific variations. Natural hybridisation, which generates phenotypic variation, has been recently explored in the triatomine group and could be more common than already recorded.^82^ ^,^ ^83^ ^,^ ^84^ ^,^ ^85^ For example, in a natural hybrid zone identified in the State of Pernambuco, Brazil, 13 phenotypes (nine of them intermediate between *T. b. brasiliensis* and *T. juazeirensis*) were revealed for *T. brasiliensis* complex, based on molecular analysis.^86^ On the other hand, the possibility of the existence of new species due to the detection of genetic variations in taxa that are now considered a single taxon, such as *Mepraia* Mazza, Gajardo & Jörg, 1940;^87^ *R. pallescens* Barber, 1932;^88^ *T. patagonica* Del Ponte, 1929;^89^ *T. costalimai* Verano & Galvão, 1958;^90^ and *R. ecuadoriensis*,^91^ ^,^ ^92^ ^,^ ^93^ ^,^ ^94^ was demonstrated.

It is crucial to highlight that in the triatomine group the descriptions of new species in the *T. brasiliensis*, *T. sordida*, *T. dimidiata*, and *Mepraia* complexes, as well as in some *Rhodnius* species, were due to integrative analysis using morphological, isoenzymatic, chromosomal and molecular studies that detected population variations compatible with the existence of species and cryptic species. A detailed comprehensive review of these cases was presented by Monteiro et al.^95^

Variety of ecotopes and the challenges of the control programs

A notorious variety of ecotopes of the triatomines described or revalidated pos-1979 was recorded. The ecotopes are in accordance with the previous knowledge of the triatomine group as mentioned in Lent & Wygodzinsky.^1^ For instance, most of the species of the *Triatoma* genus were recorded occupying rocky outcrops ecotopes, except for *T. rosai* related to distinct natural ecotopes. While *Rhodnius* species have as the primary habitat different species of palm trees, species of *Panstrongylus* genus are predominantly associated with burrows and tree cavities in their primary habitats.^32^ However, as above-mentioned, these three genera exhibit species with the ability to adapt to the anthropic environment - a process known as domiciliation.^39^ ^,^ ^96^ ^,^ ^97^

In addition to the domiciliary infestation, it is important to mention the vector control programs are not strongly supported by governmental institutions or are almost inexistent in some of the endemic countries.^98^ Another major obstacle is the interruption or reduction of *T. cruzi* transmission by native vectors.^99^ The precarious information system is also a barrier to a robust evaluation of the actual epidemiological scenario, mainly in Bolivia, Paraguay, and Mexico.^6^ In Brazil, a common problem is the lack of stable funds for vector control. Besides that, the focus to control Chagas disease is frequently weakened when other threats (e.g., dengue fever, Zika, Chikungunya, and yellow fever, and leishmaniosis) take place. Vector control strategies must be designed to overcome some of these problems, such as the Integrated Vector Management (IVM) - a worldwide trend.^7^

Furthermore, there are difficulties in monitoring and controlling the vectors in hyperendemic areas like the Gran Chaco (Argentina) because of the high infestation indices. In these areas, the use of insecticides is extremely frequent, and resistance has already been detected in *T. infestans*.^100^ More recently, localities highly infested by infected *T. brasiliensis* were also studied by Lillioso et al.^101^ raising new issues for the Northeast region, Brazil, since this species is recorded in five Brazilian states.^70^ Finally, even though there are no domiciliated species in the United States of America, an increasing number of autochthonous cases of Chagas disease has been noted, which is a matter of concern to the health authorities in that country.^102^ ^,^ ^103^

Besides the new vectorial problems, it is important to mention the threats imposed by (i) the lack of maintenance of national programs using new technologies to monitor and prevent Chagas disease;^38^ (ii) the climate change and human activities constantly changing the natural environment; and (iii) the new species of triatomines being described. This evolving scenario requires a constant monitoring activity in the endemic countries for Chagas disease, as well as comprehensive educational programs. It is now suggested that some triatomine species are able to adapt to new environmental conditions, invade new areas,^70^ and generate new phenotypes, which also poses new challenges and questions for the understanding of vector-parasite interactions and controlling of the disease, and the *T. cruzi* transmission.^86^

In conclusion

In conclusion, the 47 post-1979 triatomines described or revalidated do not seem to change the current epidemiological status of the Chagas disease, because most of them are strictly sylvatic (Table). In that list, there are only five exceptions, the first one is *T. juazeirensis*,^70^ ^,^ ^72^ ^,^ ^104^ which is very well-studied in the State of Bahia (Brazil) and frequently encountered inside houses and near forested areas; the second, also found in Bahia, is *T. sherlocki*, showing an incipient capacity for colonising domiciles.^105^ ^,^ ^106^ The third is *T. b. macromelasoma*, from Pernambuco State, where this vector is frequently found in the peridomicile however, eventually it can be found infesting the domiciles.^69^ ^,^ ^71^ The fourth one is *T. rosai* which occupies a great variety of ecotopes in several areas of Argentina, Bolivia and Paraguay^68^ ^,^ ^74^ and the fifth one is *T. huehuetenanguensis,* found naturally infected by *T. cruzi* in domestic ecotopes, being considered a potential important vector in Guatemala.^65^

Despite the evidence that the great majority of the post-1979 revalidated or new species of triatomines are not able to change the classical epidemiologic scenario of the *T. cruzi* transmission, a great effort must be devoted aiming to improve the knowledge of the recently described species. For instance, most of them lack a characterisation of their molecular profiles and even the phylogenetic relationships and detailed ecological studies. These gaps in the knowledge of a variety of species impair a more complete understanding of their evolutionary history as well as the possibility of a comparative analysis of the ecology of the Triatominae.

According to the literature, the reports of WHO^7^ and the profile of the triatomines listed in the Table, the main acknowledged vectors like *T. infestans*, *R. prolixus*, and *T. dimidiata* are going to continue to be the great threats of the *T. cruzi* transmission to human populations. Several other species presenting a more reduced geographical distribution are going to persist infesting dwellings in several countries such *as T. brasiliensis* in northeastern Brazil^70^ and *P. geniculatus* in Colômbia.^107^ In the face of the relative small epidemiologic importance of the majority of the 47 triatomines listed after Lent & Wygodzinsky,^1^ and the significant achievements in terms of modern technologies such as: diagnosis of the diseases, clinical evaluations, precise tools for molecular identification of the vector species, and the *T. cruzi* discrete typing units (DTUs) characterisation, modeling triatomines distribution throughout algorithm processes, the monitoring of vectors, and the educational programs are still the main actions to keep human populations free of Chagas disease.^108^
